# ^1^H-NMR Plasma Lipoproteins Profile Analysis Reveals Lipid Metabolism Alterations in HER2-Positive Breast Cancer Patients

**DOI:** 10.3390/cancers13225845

**Published:** 2021-11-21

**Authors:** Giuseppe Corona, Emanuela Di Gregorio, Alessia Vignoli, Elena Muraro, Agostino Steffan, Gianmaria Miolo

**Affiliations:** 1 Immunopathology and Cancer Biomarkers Unit, IRCCS Centro di Riferimento Oncologico di Aviano (CRO), 33081 Aviano, Italy; emanuela.digregorio@cro.it (E.D.G.); emuraro@cro.it (E.M.); asteffan@cro.it (A.S.); 2 Department of Molecular Science and Nano Systems, Ca’ Foscari University of Venice, Via Torino 155, Venezia Mestre, 30172 Venice, Italy; 3 Magnetic Resonance Center (CERM), Department of Chemistry “Ugo Schiff”, University of Florence, Via Luigi Sacconi 6, 50019 Sesto Fiorentino, Italy; vignoli@cerm.unifi.it; 4 Consorzio Interuniversitario Risonanze Magnetiche di Metallo Proteine, 50019 Sesto Fiorentino, Italy; 5 Medical Oncology and Cancer Prevention Unit, IRCCS Centro di Riferimento Oncologico di Aviano (CRO), 33081 Aviano, Italy; gmiolo@cro.it

**Keywords:** ^1^H-NMR, breast cancer, HER2-, lipoproteins, biomarkers, diagnosis, prognosis, metabolomics, lipidomics

## Abstract

**Simple Summary:**

Lipids’ metabolism deregulation is an established mark of breast cancer, however, no conclusive results have been reported on its effective role in disease development and progression. In this study, we applied straightforward ^1^H-NMR analysis to deeply explore alterations in circulating lipoproteins in HER2-positive breast cancer patients. The results support the key role played by lipids in the development of this breast cancer histotype and point out a specific lipid trait, characterized by a high plasma level of VLDL subfractions, potentially useful for diagnostic purposes. Moreover, the monitoring of plasma lipoproteins profile changes along the therapeutic interventions was found valuable to predict the clinical outcome.

**Abstract:**

The lipid tumour demand may shape the host metabolism adapting the circulating lipids composition to its growth and progression needs. This study aims to exploit the straightforward ^1^H-NMR lipoproteins analysis to investigate the alterations of the circulating lipoproteins’ fractions in HER2-positive breast cancer and their modulations induced by treatments. The baseline ^1^H-NMR plasma lipoproteins profiles were measured in 43 HER2-positive breast cancer patients and compared with those of 28 healthy women. In a subset of 32 patients, longitudinal measurements were also performed along neoadjuvant chemotherapy, after surgery, adjuvant treatment, and during the two-year follow-up. Differences between groups were assessed by multivariate PLS-DA and by univariate analyses. The diagnostic power of lipoproteins subfractions was assessed by ROC curve, while lipoproteins time changes along interventions were investigated by ANOVA analysis. The PLS-DA model distinguished HER2-positive breast cancer patients from the control group with a sensitivity of 96.4% and specificity of 90.7%, mainly due to the differential levels of VLDLs subfractions that were significantly higher in the patients’ group. Neoadjuvant chemotherapy-induced a significant drop in the HDLs after the first three months of treatment and a specific decrease in the HDL-3 and HDL-4 subfractions were found significantly associated with the pathological complete response achievement. These results indicate that HER2-positive breast cancer is characterized by a significant host lipid mobilization that could be useful for diagnostic purposes. Moreover, the lipoproteins profiles alterations induced by the therapeutic interventions could predict the clinical outcome supporting the application of ^1^H-NMR lipoproteins profiles analysis for longitudinal monitoring of HER2-positive breast cancer in large clinical studies.

## 1. Introduction

Cellular lipid metabolism reprogramming is a well-recognized hallmark of cancer disease [1,2,3] generally characterized by an increased lipogenesis and lipid storage, needful for the rapid tumour growth and metastatic spread [4,5,6,7]. Although de novo lipogenesis is a common marker of precursor lesions, cancer cells are also able to uptake lipids from the tumour microenvironment [8,9]. In this context, the main extra-tumour source of lipids is represented by triglycerides (TGs) derived from the surrounding adipose tissues and lipoproteins from the bloodstream [8,10]. These latter are supramolecular protein–lipids complex with a hydrophobic core of TGs and esterified cholesterol (Chol), surrounded by a double layer of phospholipids (PLs), free-Chol, and apolipoproteins (Apo) that drive their final structure and function [11]. According to density and composition, lipoproteins are classified into very-low-density (VLDL), low-density (LDL), and high-density (HDL) lipoproteins with distinct roles in lipid metabolism [12]. The VLDLs and LDLs are mainly responsible for the delivery of TGs and Chol to peripheral tissues while HDLs have a scavenger function transporting Chol and lipids from peripheral tissues to the liver [13,14,15,16,17,18].

Tumour histological types and stages can have different lipid metabolism needs and potentially can affect the levels of circulating lipoproteins [19,20,21,22,23]. So far, numerous studies have been reported to elucidate differential blood lipoproteins’ profiles in distinct cancer types [24,25,26,27]; however, heterogeneous and no conclusive findings were reported indicating that the role of lipoproteins in cancer metabolism is still far to be defined [28,29,30]. The controversial results can be partially ascribed to the commonly used clinical blood lipid profile test that is limited to the total TGs, total-Chol, Chol-HDL, and Chol-LDL. These generic classes of lipoproteins do not cover the complexity of the circulatory lipids that involve many subfractions with different lipids compositions and functions. The deep knowledge of the blood lipoproteins distribution could instead contribute to better deciphering the complex biochemical mechanisms at the base of cancer lipids metabolism deregulation occurring in cancer patients [21,30,31,32,33]. Information of such level of complexity is commonly accessible by serum/plasma lipoprotein density gradient ultracentrifugation separation followed by lipids profile analysis [34,35,36]. However, this methodological approach is very cumbersome and time-consuming and its application in clinical settings is very limited. Conversely, the proton nuclear magnetic resonance (^1^H-NMR) spectroscopy represents a high-throughput technology to study the comprehensive plasma lipoprotein profile. The ^1^H-NMR enables to distinguish the different lipoproteins subfractions by detecting changes in the magnetic chemical shift of the methyl (-CH_3_) and the methylene (-CH_2_) groups of fatty acids (FA), which is dependent on the different particle sizes as well as on the chemical heterogeneity of lipoproteins [37,38,39]. The quantification of the lipoproteins subfractions by ^1^H-NMR is currently based on the use of a validated partial least-squares regression (PLS) model obtained from the lipid region of the ^1^H-NMR spectra and ultracentrifugation lipoproteins reference data [39,40]. This emerging bioanalytical approach is an extraordinary opportunity to proficiently study the key role played by lipoprotein metabolism in cancer development and progression.

The present study aimed to explore the potential of ^1^H-NMR lipoproteins and lipids profile analysis for the discovery of potential novel diagnostic and prognostic biomarkers of HER2-positive breast cancer (BC). The preliminary results suggest that high levels of VLDL subfractions may be an important feature of HER2-positive BC patients useful for diagnostic purposes. Moreover, therapeutic interventions such as targeted neoadjuvant chemotherapy (NACT) were found to induce profound alterations in the circulating HDL lipoproteins subfractions that may be implicated in determining the response to the pharmacological treatment.

## 2. Materials and Methods

### 2.1. Patients’ Population

The study population included 43 BC patients and 28 age-matched healthy women as a control group (CTR). A subset of 32 patients was monitored during NACT, surgery, adjuvant chemotherapy treatments (ACT), and up to two-year follow-up. Participants were recruited from July 2006 to October 2010 at Centro di Riferimento Oncologico of Aviano, Italy. All the patients were diagnosed with locally advanced HER2-positive BC confirmed by histology, and all were suitable to undergo NACT consisting of six cycles of trastuzumab, loading dose 4 mg/kg intravenously, then 2 mg/kg weekly, with concomitant weekly administration of paclitaxel (80 mg/m^2^) every 21 days. The targeted pharmacological therapy was followed by surgical removal of the tumour and axillary node dissection whether sentinel lymph nodes were involved. All patients after surgery received adjuvant chemotherapy (ACT) consisting of three cycles of the paclitaxel-trastuzumab regimen followed by three months of trastuzumab administered alone at a dose of 2 mg/kg weekly.

The investigation was carried out in accordance with the principles of the Declaration of Helsinki and approved by the Ethics Committee of Centro di Riferimento Oncologico di Aviano. All subjects gave written informed consent (Clinical trial gov ID: NCT 02307227).

### 2.2. Study Design

**Figure 1:**
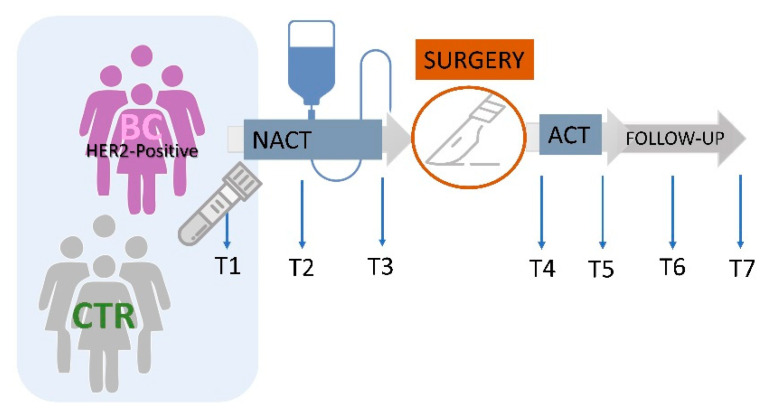
Schematic representation of the study. ^1^H-NMR lipoproteins analysis was investigated in HER2-positive BC patients (*n* = 43) at baseline (T1) and compared with those of the CTR group (*n* = 28). In a subset of patients (*n* = 32), plasma lipoproteins profiles were monitored during and after therapeutic interventions up to two-year follow-up: at baseline (T1), after three (T2) and six months (T3) from the start of NACT treatment, after two (T4) and six (T5) months from the surgery- ACT and at 1-year (T6) and two-year follow-up (T7).

This translational trial aimed at investigating the baseline status of circulatory lipoproteins in HER2-positive BC patients in comparison with an age-matched healthy group of women as well as the plasma lipoproteins profile changes along the therapeutic interventions and follow-up. Venous blood samples were collected under fasting conditions: at the diagnosis (T1), after three (T2) and six months (T3) from the NACT treatment, at two (T4) and six (T5) months from the surgery and in the first (T6) and second-year follow-up (T7) (Figure 1). The overtime variations in plasma lipoproteins fractions were investigated as a function of the achievement of the pathological complete response to NACT and to describe the differences of lipid trajectories associated with disease relapse within the 10 years of follow-ups.

### 2.3. Sample Collection

Plasma was acquired from whole blood samples collected under fasting conditions from each patient and volunteer enrolled. After collection in 5 mL tubes with anticoagulant citrate dextrose (ACD), blood samples were centrifuged at 2100 rpm for 15 min at 4 °C. The plasma in the supernatant was transferred to clean cryovials and stored at −80 °C until ^1^H-NMR analysis.

### 2.4. ^1^H-NMR Lipoproteins Analyses

Plasma lipoproteins profiles were measured by one dimensional (1D) proton ^1^H-NMR spectroscopy. All plasma samples were analysed following the standard operating procedures developed by our laboratory. Briefly, after thawing, 350 µL of each plasma sample were mixed with the same volume of a sodium phosphate buffer (70 mM Na_2_HPO_4_·7H_2_O; 6.1 mM NaN_3_; 4.6 mM sodium trimethylsilyl [2,2,3,3-^2^H_4_]-propionate (TMSP), 20% (*v*/*v*) ^2^H_2_O in H_2_O; pH 7.4). The mixture was homogenized by vortexing for 30 s. A total of 600 µL of this mixture was transferred into a 5 mm NMR tube for the NMR analysis. The ^1^H-NMR spectra were acquired at 310 K using a Bruker 600 MHz NMR spectrometer operating at 600.13 MHz proton Larmor frequency.

Lipoproteins’ subclassification and lipid quantification was performed using the Bruker IVDr Lipoprotein Subclass Analysis platform™ (B.I.-LISA, Bruker BioSpin, Singapore) that applies a validated prediction algorithm based on the PLS regression model [39,40]. The lipoproteins panel encompasses, besides the total HDL, LDL, IDL, and VLDL, 16 different subfractions numerically sort by increasing density and decreasing size: four HDL-subfractions (HDL-1 to HDL-4), six LDL subfractions (LDL-1 to LDL-6), six VLDL subfractions (VLDL-1 to VLDL-6). For each subfraction, Chol, free-Chol, PLs, TGs, Apo A1, A2, B100 fractions were quantified together with 10 particle numbers and Chol-LDL/Chol-HDL and Apo-B100/Apo-A1 ratios for a total of 112 parameters (Table S1).

### 2.5. Statistical Analyses

Quantitative lipoproteins profile data from ^1^H-NMR spectra were pre-processed by log transformation and Pareto-scaled before statistical analyses. Multiparametric supervised PLS-DA was used to determine differences in lipoproteins subtractions parameters between HER2-positive BC and CTR groups as well as to find associations with the pathological complete response to the NACT. The PLS-DA models were validated using K-fold cross-validation (CV), splitting the dataset into seven different subsets and using the six subsets as a training set and the left single subset for testing the model. The quality parameters R^2^ and Q^2^ were used to evaluate the goodness of fit and the predictive ability of the model, respectively. A permutation test was carried out to exclude data over-fitting while the cross-validated residuals ANOVA was performed to assess PLS-DA model reliability. Significant different lipoprotein subfractions were selected by variable Importance in Projection (VIP) and by univariate Student’s *t*-test. False discovery rate (*q*) based on multiple comparison procedures was assessed to control simultaneous statistical inferences. A statistically significant variable was considered for a *q* < 0.05 unless otherwise specified. Receiver operating characteristic (ROC) analyses were applied to determine the diagnostic power of the selected plasma lipoproteins concentrations for differentiating the groups of interest. The time-related trends in the lipoprotein subfractions along therapeutic interventions and follow-up were investigated by one-way analysis of variance (ANOVA) followed by Tukey’s post hoc. Time-dependent plasma lipoproteins trajectories were obtained for patients with and without recurrence, plotting the mean scores of the first two components of the principal component analysis (PCA) model of plasma lipoproteins evaluated at each time-point from T4 to T7 [41]. Data statistical analyses were carried out using SIMCA (Umetrics, v. 14.1) software and Metaboanalyst v. 4.0 [42].

## 3. Results

### 3.1. Study Populations’ Characteristics

The 43 HER2-positive BC and 28 healthy women enrolled in the study showed homogeneous demographic characteristics (Table 1). The median age of the patients and CTR group was 49 years (range: 23–70) and 50 years (range: 28–70), respectively (*p* = 0.905). The two groups did not differ for the body mass index (BMI) (*p* = 0.801) with a prevalence of normal weight (BMI < 25) in both groups corresponding to 64.7% of the BC patients and 70.1% in the CTR population, respectively. Pre-menopausal women accounted for 58.1% of BC patients and for 57.1% of controls, respectively, while the remaining 41.9% of BC patients and 42.9 of controls were in post-menopausal status (*p* = 1, Fisher exact test). At the diagnosis, 76.7% of the HER2-positive BC presented stage II disease, while the remaining were classified at stage III. Over half (53.5%) of BC tumours were characterized by a highly proliferative tumour index with a Ki67 ≥ 20, while 46.5% had a less aggressive phenotype. A little more than half (51.2%) of patients carried oestrogen receptor (ER) positive tumours. All patients underwent NACT based on trastuzumab-paclitaxel followed by surgery. The 56.2% of patients achieved pathological complete response (CR) after treatments while the remaining ones reported only a partial response (PR). Over the 10 years of follow-ups, the disease relapse occurred in 28.1% of the patients, where the majority belonged to the ER-positive subtype (89%).

### 3.2. Lipoproteins Subfractions Profile in HER2-Positive BC Patients vs. CTR Group

**Figure 2:**
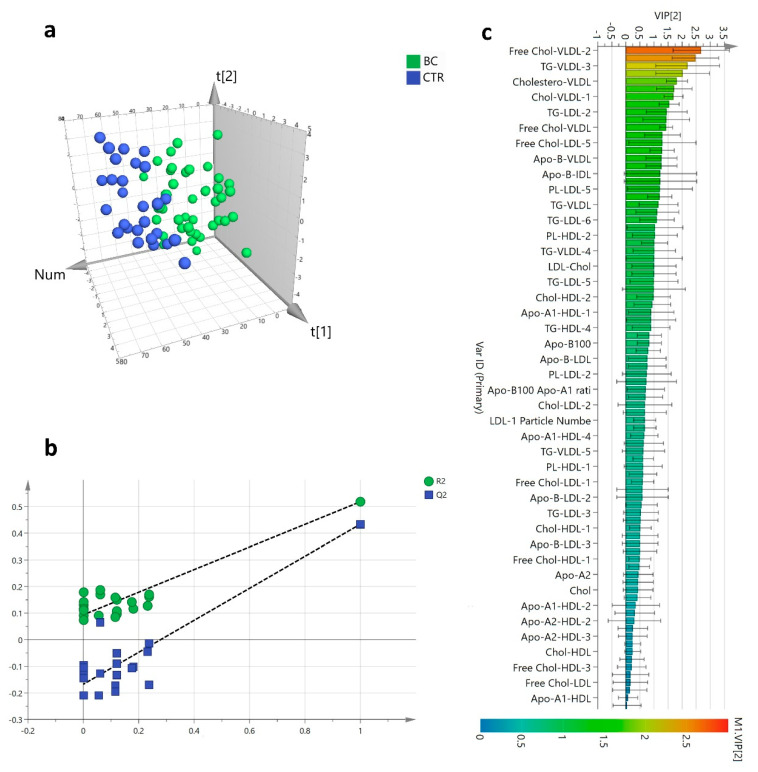
Plasma lipoproteins profiles PLS-DA score plot of HER2-positive BC patients (*n* = 43) and the CTR group (*n* = 28) (**a**); Permutation test plot showing R^2^ (green) and Q^2^ (blue) values from the permuted models (**b**); Variable importance plot for the projection (VIP) score of the PLS-DA model ranked by increasing values (**c**).

**Figure 3:**
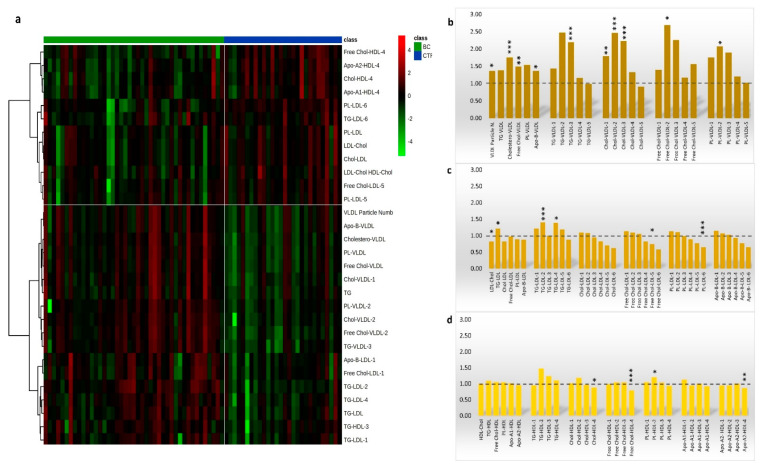
Heat maps of ^1^H-NRM lipoproteins subfractions significantly differed between HER2-positive BC patients and the CTR group. High values are coloured in red while low values in green with a different brightness according to the magnitude of the difference when compared with the average value (**a**). Mean fold change in BC referred to CTR of the VLDL (**b**), LDL (**c**), HDL (**d**) main fractions, and subfractions. *** *q* < 0.001; ** *q* < 0.01; * *q* < 0.05.

The ^1^H-NMR plasma lipoproteins profile targeted 112 lipoproteins’ parameters of which 101 (90%) were quantified while 11 (10%) were excluded being out of range of the validated assay (Table S1). The supervised partial least squares discrimination analysis (PLS-DA) showed that HER2-positive BC patients had a spatial distribution significantly different from that of the CTR group (Figure 2a) The classification model had good reliability (R^2^ = 0.79, Q^2^ = 0.47) yielding 90.7% of sensitivity and 92.9% of specificity without any potential risk of over-fitting (Figure 2b). The lipoproteins’ subfractions that significantly contributed to group separation (VIP ≥ 1) belonged mainly to the VLDLs and LDLs (Figure 2c). The relative concentration distributions of such lipoproteins between the two groups are reported in a heatmap (Figure 3a) together with the fold-change referred to the CTR group (Figure 3b–d).

The plasma level of overall VLDL resulted significantly increased in HER2-positive BC patients, in particular for their total Chol-VLDL (*p* = 6.9 × 10^−5^; *q* = 0.001), free-Chol (*p* = 0.001; *q* = 0.006) and PLs (*p* = 3.7 × 10^−4^; *q* = 0.004) contents (Table S2). Among VLDL subfractions, significant differences were observed in particular for the VLDL-1, VLDL-2, and VLDL-3 whose Chol contents resulted in 1.8, 2.5, and 2.2-fold higher in BC compared with the CTR group respectively (Figure 3b, Table S2). The LDLs were found slightly low in HER2-positive BC patients especially the LDL-4, LDL-5, and LDL-6 carrying free-Chol, PL, and ApoB (Figure 3c, Table S2) while a significant decrease was observed for the PL-LDL-6 (*p* = 4.9 × 10^−6^; *q* = 2.1 × 10^−4^) and the free- Chol-LDL-5 (*p* = 0.012; *q* = 0.049) subfractions (Table S2). Conversely, the TGs contents of LDL-2 and LDL-4 resulted in significant increases in BC patients as well as the total TGs (*p* = 0.005; *q* = 0.023). Unlike VLDLs and LDLs the plasma HDLs were generally poorly affected by the presence of the HER2-positive BC (Figure 3d), except for the content of free Chol (*p* = 6.5 × 10^−5^; *q* = 0.001), Chol (*p* = 0.002; *q* = 0.013) and Apo A2 (*p* = 0.001; *q* = 0.008) in HDL-4 that resulted significantly decreased in BC patients (Table S2). The plasma lipoproteins profile was also investigated according to menopausal status. Within the BC group, the post-menopausal patients showed higher baseline HDL and LDL subfractions compared with pre-menopausal ones, while for the VLDLs subfractions no differences were observed (Figure S1). Moreover, no alterations in baseline circulatory lipoproteins were found associated with the tumour’ ER status since the PLS-DA showed many overlaps between ER-positive and ER-negative patients (Figure S2).

### 3.3. Lipoproteins Profile and HER2-Positive BC Diagnosis

**Figure 4:**
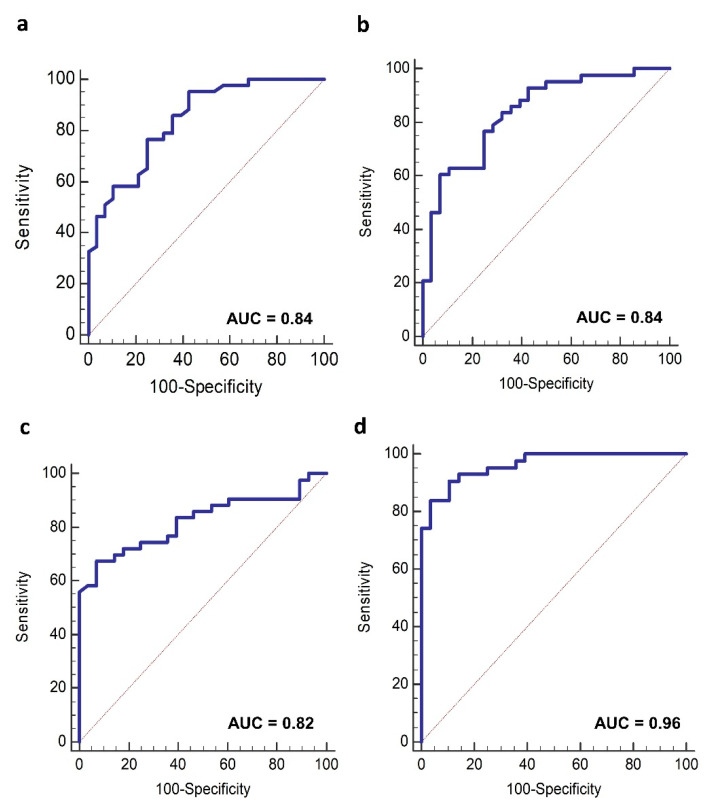
Receiver operating characteristic (ROC) curve analysis of the lipoproteins subfractions that best discriminate HER2-positive BC patient from CTR: free-Chol VLDL-2 (**a**); Chol-VLDL-2 (**b**); PL LDL-6 (**c**) single variables and their ROC model combination (**d**).

The potential diagnostic value of baseline levels of VLDL lipoproteins subfractions for distinguishing HER2-positive BC patients from healthy CTR was investigated by ROC analyses. The highest AUC was obtained for the free Chol-VLDL-2 (AUROC = 0.84, 95% CI 0.75–0.93), Chol-VLDL-2 (AUROC = 0.84, 95% CI 0.73–0.92), and PL-LDL-6 (AUROC = 0.82, 95% CI 0.71–0.90) (Figure 4a–c). The combination of these lipoproteins subtractions was found to increase the AUC to 0.96 (95% CI 0.89–0.99) with a sensitivity and specificity of 83.72% and 96.43%, respectively (Figure 4d).

### 3.4. Lipoproteins Profiles Time Changes

**Figure 5:**
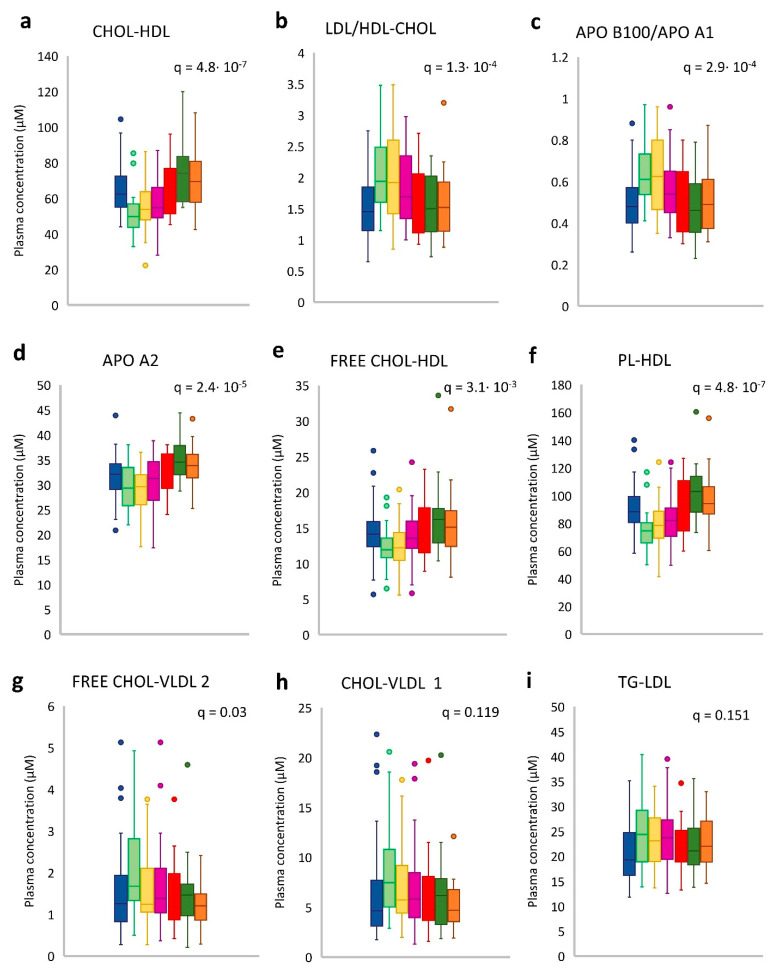
Time-related changes of some representative plasma lipoproteins levels over the treatments and follow-up (*n* = 32). Boxes are coloured differently accordingly to the time point: the diagnosis (T1, blue), after three (T2, light green) and six months (T3, yellow) from the NACT starting, two months (T4, violet), and six (T5, red) months after the surgery, in the first-year (T6, dark green) and two-year follow-up (T7, orange). Horizontal lines indicated the median values that separated the lower 25th and the upper 75th quartile; vertical lines denote the highest and lowest whiskers; dots are observations outside the quartiles range.

The plasma ^1^H-NMR lipoproteins profiles were monitored over time in a subgroup of 32 HER2-positive BC patients to highlight the effect of treatments on circulatory lipids and lipoproteins status. The ANOVA analysis showed that the HDL was the class mainly affected by the pharmacological intervention (Table S3). Almost all the lipids and apolipoproteins belonging to the HDL class underwent a significant drop during the first 3 months (T2) of NACT. The principal alterations regarded the Chol-HDL (*p* = 1.4 × 10^−8^; *q* = 4.8 × 10^−7^), Apo A2 (*p* = 2.5 × 10^−6^; *q* = 2.4 × 10^−5^), free Chol-HDL (*p* = 6.2 × 10^−4^; *q* = 3.1 × 10^−3^) and PLs-HDL (*p* = 8.8 × 10^−9^; *q* = 4.8 × 10^−7^) (Table S3). In agreement with such specific and selective HDLs drop, the Chol-LDL/Chol-HDL ratio, as well as their respective Apo B100/ApoA1 ratio, showed marked increases during the first 6 months of NACT that were restored to baseline levels only at the end of the two-year follow-up (Figure 5b,c). Among the other lipoproteins classes, only the free Chol-VLDL-2 (*p* = 0.008; *q* = 0.033) increased during paclitaxel–trastuzumab treatment (Figure 5g) together with the Chol-VLDL-1 (*p* = 0.031; *q* = 0.119) (Figure 5h), and total TG-LDL (*p* = 0.042; *q* = 1.372), (Figure 5i), however these latter did not retain the statistically significance after the correction for multiple tests. Surgery only partially affected the lipoproteins profile since non-significative differences can be reported from T3 and T4 time points. Interestingly, the same dose of paclitaxel-trastuzumab during the first two months of ACT (T4) does not replicate the HDLs drops early observed during NACT but from T2 up to T7, it was instead reported a gradual increase above the baseline levels (Figure 5a,d–f).

**Figure 6:**
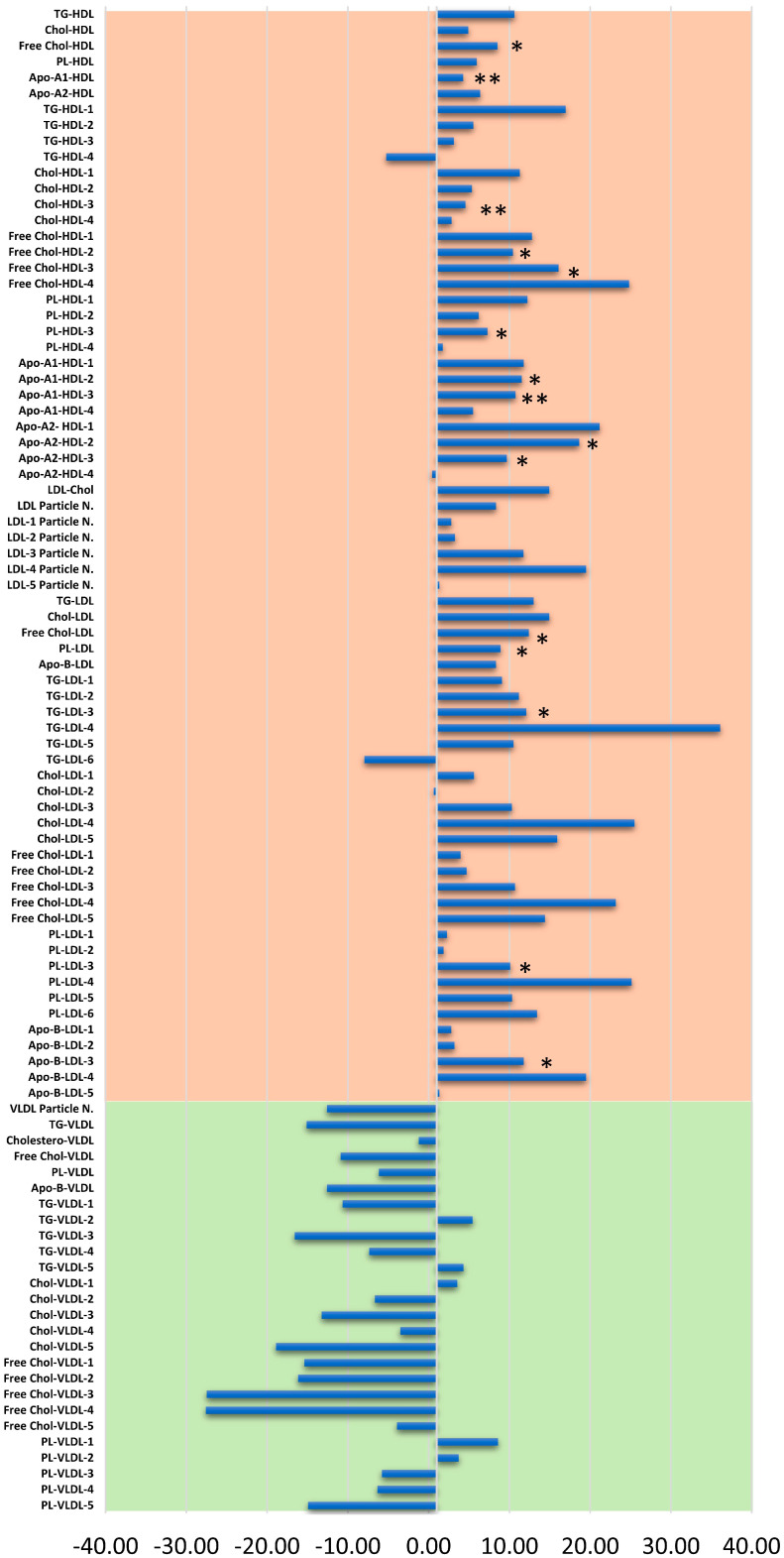
Changes in plasma lipoproteins concentrations after the two-year follow-up referred to baseline expressed as percentage relative differences. ** *p* < 0.01; * *p* < 0.05.

At the end of the two years of follow-ups (T7), almost all the HDL and LDL subfractions resulted in a slight increase of about 6.9% (range, −5.3–24.9%) and 10.3% (range, −7.9–30.1%), respectively as compared with baseline levels; conversely, the majority of the VLDL subfractions had lower results than the initial condition with a relative percentage difference of −9.01% (range, −27.59–8.58%) though not reaching statistical significance (Figure 6).

### 3.5. Lipoproteins Changes and Clinical Outcome

**Figure 7:**
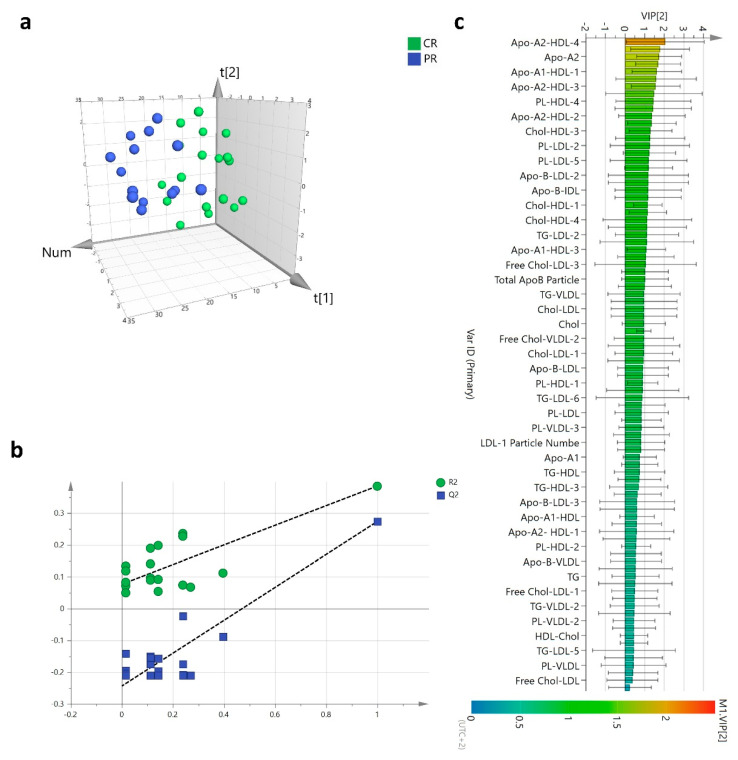
PLS-DA discriminated plasma lipoproteins profiles of patients achieving a CR (*n* = 18), green) and a PR (*n* = 14, blue) (**a**); Permutation test plot showing R^2^ (green) and Q^2^ (blue) values from the permuted models (**b**); Variables importance for the projection (VIP) score plot of the PLS-DA model (**c**).

The response to the NACT treatment was not homogeneous among patients. The best response consisted in the achievement of pathological CR which was reported in 18 patients while the remaining 14 experienced a PR with the residual presence of cancer cells. The main alterations of plasma lipoproteins occurred after the first three months of NACT treatment and were found associated with the pharmacological outcome. The PLS-DA of lipoproteins changes at three months clearly differentiated the CR from the PR groups (*p* = 0.05, CV-ANOVA) (Figure 7a).

**Figure 8:**
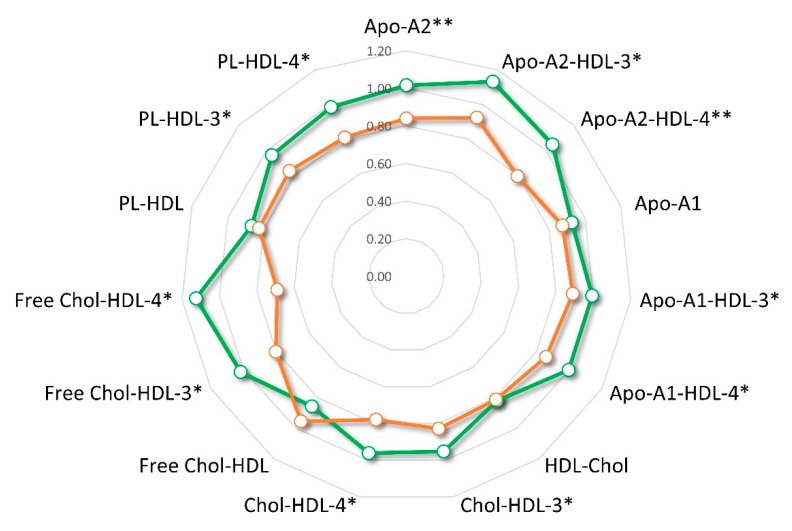
Radar plot of plasma HDL subfractions’ changes after 3 months (T2) of NACT as compared with baseline (T1) in complete (CR, green) and partial response (PR, red) patients. ** *p* < 0.01; * *p* < 0.05.

The PLS-DA model showed acceptable performance (R^2^ = 0.75, Q^2^ = 0.27) without any potential risk of over-fitting (Figure 7b). The most relevant lipoproteins involved in the group separation (VIP ≥ 1) belonged mainly to the HDL class which had significantly lower results in the PR group compared with the CR (Figure 7c). In particular, the plasma reduction interested HDL-3 and HDL-4 subfractions and their Apo-A1, Apo-A2, and as well as their lipid contents with the exclusion of TGs. The mean overall fold change at three months of NACT treatment when referred to the baseline was 0.83 (range, 0.69–0.95) in PR patients, significantly lower than 0.98 (range, 0.82–1.13) (*p* = 2.9 × 10^−5^) reported in CR patients (Figure 8).

### 3.6. Plasma Lipoproteins Trajectories and Recurrence

**Figure 9:**
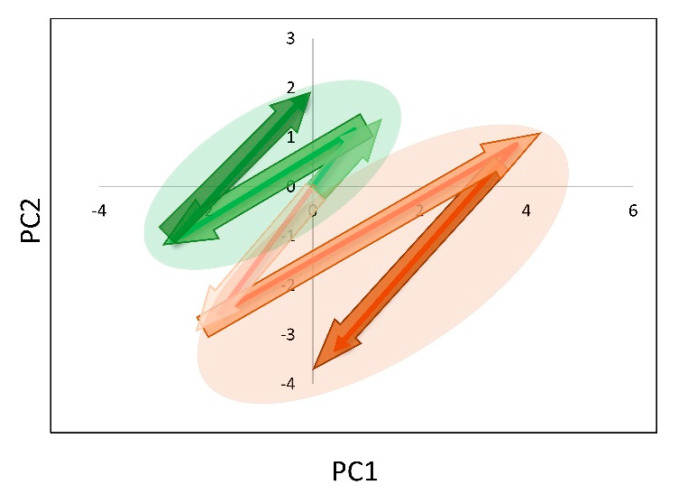
Lipoproteins profile trajectories during the two-year follow-up from T4 to T7 of relapsed and disease-free ER-positive patients. Trajectories were evaluated by using the first two components score from PCA analysis performed at each time point and normalized to T4 value. Trajectories of relapsed (*n* = 8) and disease-free (*n* = 10) patients are coloured in red and green, respectively.

In over 10 years of clinical follow-ups, disease recurrence was reported in 9 (28%) patients. All the events occurred after the two years of the study’s timeframe. Among the relapsed patients, eight had an ER-positive BC subtype, thus, to avoid any potential confounding effect due to ER status, only ER-positive patients were considered in the analysis. The plasma lipid trajectories of relapsed patients (*n* = 8)—evaluated by PCA from T4 to T7 timeframe—were characterized by an opposite direction to those of disease-free patients (*n* = 10) (Figure 9).

## 4. Discussion

In this study, we explored the ^1^H-NMR potential to characterize the plasma lipoproteins profiles of a population of BC patients homogeneous for HER2-hystotype. The comparison with a CTR group pointed out that the HER2-positive BC patients showed at diagnosis significant higher plasma concentrations of specific VLDL subfractions carrying Chol and free-Chol (Figure 3). Previous epidemiological studies have shown a relationship between BC and lipid disorders that included elevated Chol-VLDL [29,43] and Chol-LDL [29,44,45,46] as well as total Chol [44,47,48], often associated with decreased Chol-HDL [44,47,48,49] all characteristics of obesity. In the present investigation, no specific physical and clinical data were available to precisely estimate the individual visceral adiposity or the presence of metabolic syndromes. However, BMI values of all patients were in the normal range, suggesting that the increase of their circulatory VLDL levels might be likely associated with the tumour lipid reprogramming metabolism. This hypothesis finds support from previous studies where blood Chol level was reported to increase in BC patients irrespective of their BMI or metabolic syndrome [50,51]. On this basis, BC per se may cause the blood Chol rise, although other studies reported opposed evidence [22,52]. These inconsistent results could be due to the lack of knowledge about the Chol distribution among the different lipoproteins subfractions that limit the conclusive interpretation. In the present investigation, the deep and straightforward lipid analysis allowed by the ^1^H-NMR platform has revealed that HER2-positive BC may induce lipid alterations of specific Chol-VLDL subfractions such as the low size VLDL-1,2,3 which are commonly disregarded by routine clinical lipid tests. The increased Chol mobilization through the specific VLDL-1,2,3 subfractions may represent a distinctive feature of HER2-positive BC underlining the potential key role played by circulation lipoproteins in the disease development. Interestingly, the best diagnostic ROC model was based only on such specific VLDL subfractions excluding the conventional total Chol, Chol-VLDL, Chol-LDL, and Chol-HDL incorporated in clinical practice, further supporting the potentiality of specific lipoproteins subfractions for diagnostic purposes.

The HER2-positive BC group accounts for approximately 15–25% of all BCs, and it is characterized by high tumour growth rates and aggressive clinical behaviour [53] linked also to cellular lipids deregulation [44,54,55]. The HER2 expression-mediated signalling enhances the activation of specific metabolic networks such as the PI3K/AKT/mTOR pathway hyperactivation, which leads to the up-regulation of both glycolysis and FA synthase enzymes [6,54,55,56,57,58]. Besides this lipogenesis phenotype, HER2-tumour cells are also characterized by an increased exogenous FA uptake from the surrounding microenvironment by lipoprotein lipase activity and by the membrane expression of CD36 and LDL receptors (LDLR) [10,59,60,61]. LDLR hyperexpression in HER2-positive tumour cells and triple-negative BC phenotypes were found associated with increased invasion [62,63], while the LDLR downregulation in BC cells was reported to enhance cancer cell death and reduce the tumour growth in the BC hyperlipidaemia model [64,65].

The VLDL subfractions are mainly involved in the lipid delivery to peripheral tissues and their high availability in the plasma of HER2-positive BC patients may represent a specific high-risk phenotype independently of the menopausal status. This hypothesis is in agreement with the findings of some previous investigations which reported serum TGs and Chol-VLDL significantly increased in advanced HER2-positive and triple-negative BC [21,22,29,55]. Conversely, a recent study reported an inverse association between some VLDL subfractions and the BC developing risk suggesting a potential protective effect of such lipoproteins class especially in pre-menopausal BC patients [66]. However, it is worth noting that this finding arises from a study involving heterogeneous BC subtypes thus not excluding that VLDLs, in HER2-positive BC, may play a different role.

The VLDL subfraction rise in HER2-positive BC could be related to the tumour effect on the host lipid metabolism. An elegant research study in animal models demonstrated that cancer itself is effectively able to induce hyperlipidaemia by enhancing VLDL production and impairing VLDL/LDL turnover [67]. Thus, the observed high systemic level of almost all VLDL subfractions in the HER2-positive BC patients may be the result of a liver biosynthetic rebound induced by tumour lipid shortage shifting circulating lipoproteins homeostasis. Beyond VLDLs, also the total TGs and some TGs enriched lipoproteins subfractions were significantly higher in HER2-positive BC patients in agreement with a recent investigation where elevated blood TGs concentrations were linked to an inflammatory status and a greater BC risk [68]. All these results taken together, appear to sustain the hypothesis that a greater host lipid mobilization may have a noteworthy effect on tumour proliferation. Deviations from plasma lipoproteins homeostasis were also observed along the therapeutic interventions consisting of the NACT followed by the surgical removal of the residual tumour. After three months from treatment, the HDL lipoproteins, in particular, subfractions Apo-A1 and Apo-A2, PLs, Chol, and free-Chol underwent a significant plasma drop (Figure 5). Such specific ApoA-HDL shortage induced by NACT was also reported in other investigations during both neoadjuvant and adjuvant BC treatments that incorporated cytotoxic drugs such as anthracycline and paclitaxel [45,69,70,71]. The HDL subfractions decrease seems to be linked to the drug’s insult at the liver site where HDL biosynthesis occurs. This hypothesis is supported by in vitro studies that report chemotherapy agents able to down-regulate the cellular liver expression of the peroxisomal proliferator-activated receptor γ (PPARγ), liver X receptor α (LXRα), and the ATP binding cassette transporter A1 (ABCA1) [70,72], all involved in lipoproteins metabolism. The chemotherapy effect on HDL Apo-A1, Apo-A2, and lipid subfractions was transient since their levels were almost restored after the chemotherapy cycles. However, the extent variation of HDL-3 and HDL-4 subfractions at three months resulted interestingly associated with a poor pharmacological outcome (Figure 8). The HDL-3 subfraction, besides its common function as reverse cholesterol transporter mediated by ABCA1 carrier [73], was found to prevent lipid oxidation by the redox activity of Apo-A and other enzymes [74,75] present in small dense HDL-3 and HDL-4 subfractions [76,77,78]. Such antioxidant properties could be relevant to protect against the chemotherapy-associated oxidative stress since the patients that experienced wide HDL-3 subfractions loss, may have a minor ability to contrast the drug’s side effects, compromising optimal response to NACT. Moreover, specific HDL-Apos are involved in the innate and adaptive immune responses primarily through the modulation of lipid raft components in monocytes/macrophages, dendritic cells, and T and B lymphocytes [79,80,81]. Such function is important in determining the antibody-dependent cellular cytotoxicity triggered by the binding of trastuzumab to HER2-Receptor [82] and in this context, the low plasma HDL-3 and HDL-4 levels could reflect a compromised immunophenotype unable to respond to the immune stimulation of the treatment.

After two months from surgery and ACT with paclitaxel-trastuzumab, the plasma concentrations of almost all lipoproteins’ subfractions did not significantly differ from those measured at the end of NACT. The lack of the HDLs drop along the ACT can be likely ascribed to the maintenance of the homeostasis biochemical mechanisms altered by the previous NACT treatment. Thus, from T2, after surgery and adjuvant treatments, an overall slight increase trend for HDL was observed up to the two years follow-up similar to that reported in other studies [83,84]. Interestingly, conversely to HDLs, at the end of the two-year follow-up, the VLDLs (Figure 6) were found all almost reduced as compared with the baseline levels suggesting that the therapeutic intervention could partially reset the high cancer VLDL phenotype observed at diagnosis. However, the VLDL reduction resulted only partially, not reaching the level observed in the CTR indicating that the abnormal lipids mobilization induced by the HER2-positive BC may require much more time for its normalization, thus, indicating that cancer may strongly modify the host metabolism far away from its homeostasis status.

During 10 years of follow-ups, eight ER-positive BC patients relapsed. These patients, representing about 89% of disease recurrences, showed different and divergent lipoprotein subfractions profile trajectories compared with disease-free patients (Figure 9). The divergent lipid trajectories observed in this study may be suggestive of a distinctive dynamic lipid pattern for the relapsed patients. This hypothesis was similarly reported by Shah et al. [54] where different trends for total Chol, Chol-LDL, TG, and Chol-VLDL were observed in relapsed/metastatic patients and in disease-free patients.

To our knowledge, the present study represents the first investigation attempted to examine the role of circulating lipoprotein profile in a uniform BC population of HER2-positive patients and all undergoing the same NACT treatment.

Despite this strong point, the study still presents the limitation of the low sample size due to the rarity of the HER2-positive BC histotype. Such low numerosity along with the lack of information about patients’ lifestyle and comorbidities, limits to drawing definitive conclusions about the role of VLDL subfractions in combination with other cancer risk factors and impose further clinical validation of the results in an independent and larger population of patients.

## 5. Conclusions

The results of this explorative study sustain the presence of lipids dysregulation trait in HER2-positive BC. The level of knowledge on circulatory lipoproteins subfractions offered by ^1^H-NMR allowed for pointing out specific high levels of VLDL subfractions in HER2-positive BC patients. This specific VLDL signature may be relevant to shed new light on the reprogramming process of the host lipid metabolism induced by HER2-positive BC development and it can be useful to enhance the early detection of this disease. The longitudinal monitoring of plasma lipoproteins profile can be useful to predict the outcome of the therapeutic interventions supporting the application of ^1^H-NMR lipoproteins analysis to better identify specific lipid trajectories of released patients in large longitudinal clinical investigations.

## Figures and Tables

**Table 1:** Demographic and clinical characteristics of the HER2-positive BC patients and the CTR group.

Characteristics	BC (*n* = 43)
Age, years (Median, range)	49 (23–70)
BMI, kg/m^2^ (Mean ± SD)	25.0 ± 5.3
Pre-menopause (*n*, %)	25 (58.1)
Post-menopause (*n*, %)	18 (41.9)
Stage (*n*, %)	
II	33 (76.7)
III	10 (23.3)
Ki67 (*n*, %)	
<20	20 (46.5)
≥20	23 (53.5)
ER status (*n*, %)	
Positive	22 (51.2)
Negative	21 (48.8)
Pathological Response (*n*, %) *	
Complete	18 (56.2)
Partial	14 (43.8)
Recurrence (*n*, %) *	
Yes	9 (28.1)
No	23 (71.9)

* referred to *n* = 32 subset of patients in the longitudinal study.

## Data Availability

The data presented in this study are available on request from the corresponding author. The data are not publicly available due to privacy.

## Supplementary Materials

The following are available online at https://www.mdpi.com/article/10.3390/cancers13225845/s1, Table S1: List of lipoproteins measured by ^1^H NRM, Table S2: Lipoproteins significantly altered in BC patients and in the CTR group baseline plasma, Table S3: Significant time-related changes of lipoproteins by ANOVA, Figure S1: Plasma lipoproteins profiles PLS-DA score plot of HER2-positive BC patients in pre-menopause and post-menopause, Figure S2: PLS-DA of the plasma baseline lipoproteins’ profile for BC patients stratified according to the ER status.
