# Supplementary material for: 1H-NMR Plasma Lipoproteins Profile Analysis Reveals Lipid Metabolism Alterations in HER2-Positive Breast Cancer Patients
Source: Cancers (Basel). 2021 Nov 21;13(22):5845. doi: 10.3390/cancers13225845 (PMC8616511; doi:10.3390/cancers13225845)
Supplement: Supplementary file 1 [file cancers-13-05845-s001.zip › cancers-1451612-supplementary.pdf]

cancers

MDPI

Article

^{1}H-NMR plasma lipoproteins profile analysis reveals lipid metabolism alterations in HER2-positive breast cancer patients

Giuseppe Corona[1§*], Emanuela Di Gregorio[1,2§], Alessia Vignoli[4,5], Elena Muraro[1], Agostino Steffan[1] and Gianmaria Miolo[3]

[1] IRCCS Centro di Riferimento Oncologico di Aviano (CRO), Immunopathology and Cancer Biomarkers Unit, 33081 Aviano, Italy; emanuela.digregorio@cro.it (E.D.G.); emuraro@cro.it (E.M.); asteffan@cro.it (A.S.)
[2] Ca' Foscari University of Venice, Department of Molecular Science and Nano Systems, Via Torino 155, Venezia Mestre, 30172 Venice, Italy
[3] Magnetic Resonance Center (CERM), Department of Chemistry "Ugo Schiff", University of Florence, Via Luigi Sacconi 6, 50019 Sesto Fiorentino, Italy; vignoli@cerm.unifi.it
[4] Consorzio Interuniversitario Risonanze Magnetiche di Metallo Proteine, 50019 Sesto Fiorentino, Italy
5 IRCCS Centro di Riferimento Oncologico di Aviano (CRO), Medical Oncology and Cancer Prevention Unit, 33081 Aviano, Italy; gmiolo@cro.it
* Correspondence: giuseppe.corona@cro.it; Tel.: +39-0434-659-666

§ Equal contributions
* Correspondence: giuseppe.corona@cro.it (G.C.); Tel.: +39-0434-659666 (G.C.)

Table S1. List of lipoproteins measured by ^{1}H NRM

	TGs	Chol	Free Chol	PLs	Apo-A1	Apo-A2	Apo-B	P.N.
VLDL	✓	✓	✓	✓	–	–	✓	✓
IDL	✓ (red)	✓ (red)	✓ (red)	✓ (red)	–	–	✓	✓ (red)
LDL	✓	✓	✓	✓	–	–	✓	✓
HDL	✓	✓	✓	✓	✓	✓	–	–
VLDL 1	✓	✓	✓	✓	–	–	✓	–
VLDL 2	✓	✓	✓	✓	–	–	✓	–
VLDL 3	✓	✓	✓	✓	–	–	✓	–
VLDL 4	✓	✓	✓	✓	–	–	✓	–
VLDL 5	✓	✓	✓	✓	–	–	✓	–
VLDL 6	✓	✓	✓	✓	–	–	✓	–
LDL 1	✓	✓	✓	✓	–	–	✓	✓ (red)
LDL 2	✓	✓	✓	✓	–	–	✓	✓ (red)
LDL 3	✓	✓	✓	✓	–	–	✓	✓ (red)
LDL 4	✓	✓	✓	✓	–	–	✓	✓ (red)
LDL 5	✓	✓	✓	✓	–	–	✓	✓ (red)
LDL 6	✓	✓	✓	✓	–	–	✓	✓ (red)
HDL 1	✓	✓	✓	✓	✓	✓	–	–
HDL 2	✓	✓	✓	✓	✓	✓	–	–
HDL 3	✓	✓	✓	✓	✓	✓	–	–
HDL 4	✓	✓	✓	✓	✓	✓	–	–

TGs, tryglicerides; Chol, cholesterol; Apo, apolipoprotein; PLs, phospholipids; P.N, particle numbers; ✓ (red), detected but not quantified; ✓, detected and quantified.

Table S2. Lipoproteins significantly altered in BC patients and in the CTR group baseline plasma.

	HER2 (+) BC Mean ± SE (n = 43)	CTR Mean ± SE (n = 28)	p- value	q-value
**TG**	105.4 ± 8.3	76.4 ± 6.1	0.005	0.024
**Free Chol-VLDL-2**	1.5 ± 0.2	0.5 ± 0.1	4.1E-07	3.6E-05
**Chol-VLDL-2**	3.9 ± 0.4	1.6 ± 0.2	2.4E-05	4.7E-04
**TG-VLDL-3**	10.4 ± 1.0	4.7 ± 0.6	2.8E-05	4.7E-04
**Chol-VLDL**	20.5 ± 1.9	11.6 ± 1.4	6.9E-05	0.001
**PL-VLDL**	19.8 ± 1.5	12.9 ± 1.3	3.7E-04	0.004
**Chol-VLDL-1**	6.3 ± 0.7	3.5 ± 0.5	4.2E-04	0.004
**Free Chol-VLDL**	9.1 ± 0.7	6.1 ± 0.6	0.001	0.006
**PL-VLDL-2**	3.1 ± 0.3	1.5 ± 0.2	0.002	0.014
**VLDL Particle N.**	130.5 ± 10.4	95.7 ± 9.7	0.012	0.049
**Apo-B-VLDL**	7.2 ± 0.6	5.3 ± 0.5	0.012	0.049
**PL-LDL-6**	8.8 ± 0.7	13.7 ± 0.7	4.9E-06	2.1E-04
**TG-LDL-2**	2.9 ± 0.1	2.1 ± 0.1	1.1E-05	3.0E-04
**Chol-LDL**	93.6 ± 3.9	114.3 ± 5.7	0.003	0.017
**TG-LDL-4**	2.9 ± 0.2	2.1 ± 0.2	0.008	0.037
**Free Chol-LDL-5**	3.5 ± 0.2	4.7 ± 0.4	0.012	0.049
**TG-LDL**	20.8 ± 0.9	17.2 ± 1.1	0.012	0.049
**Free Chol-HDL-4**	3.3 ± 0.1	4.2 ± 0.2	6.5E-05	0.001
**Apo-A2-HDL-4**	18.6 ± 0.5	21.6 ± 0.7	0.001	0.008
**Chol-HDL-4**	21.1 ± 0.6	23.8 ± 0.7	0.002	0.013
**PL-HDL-2**	14.1 ± 0.6	13.4 ± 0.2	0.005	0.024

SE, standard error; q-value= FRD

Table S3. Significant time-related changes of lipoproteins by ANOVA*

Name	p- value	q- value	T1	T2	T3	T4	T5	T6	T7
Chol-HDL	1.41E-08	4.80E-07							
Free Chol-HDL	0.001	0.003							
Chol-LDL/Chol-HDL	2.01E-05	1.35E-04							
Apo-B100/Apo-A1	4.81E-05	2.89E-04							
PL-HDL	8.77E-09	4.80E-07							
Apo-A1-HDL	1.68E-08	4.80E-07							
Apo-A2-HDL	3.68E-06	3.02E-05							
Chol-HDL-1	7.77E-07	9.84E-06							
Chol-HDL-2	3.70E-06	3.02E-05							
Chol-HDL-3	1.82E-05	1.30E-04							
Chol-HDL-4	3.99E-04	0.002							
Free Chol-HDL-1	0.001	0.004							
Free Chol-HDL-2	0.005	0.020							
Free Chol-HDL-3	3.43E-04	0.002							
PL-HDL-1	5.62E-07	8.38E-06							
PL-HDL-2	1.85E-06	1.91E-05							
PL-HDL-3	5.88E-07	8.38E-06							
PL-HDL-4	1.52E-04	0.001							
Apo-A1-HDL-1	1.64E-06	1.87E-05							
Apo-A1-HDL-2	3.64E-07	6.91E-06							
Apo-A1-HDL-3	1.72E-05	1.30E-04							
Apo-A1-HDL-4	0.001	0.005							
Apo-A2- HDL-1	0.005	0.020							
Apo-A2-HDL-2	4.62E-05	2.89E-04							
Apo-A2-HDL-3	0.001	0.006							
TG-LDL	0.042	0.151							
Chol-VLDL-1	0.031	0.119							
Free Chol-VLDL-2	0.008	0.033							

*Grading colors from green to black to red indicate increasing value

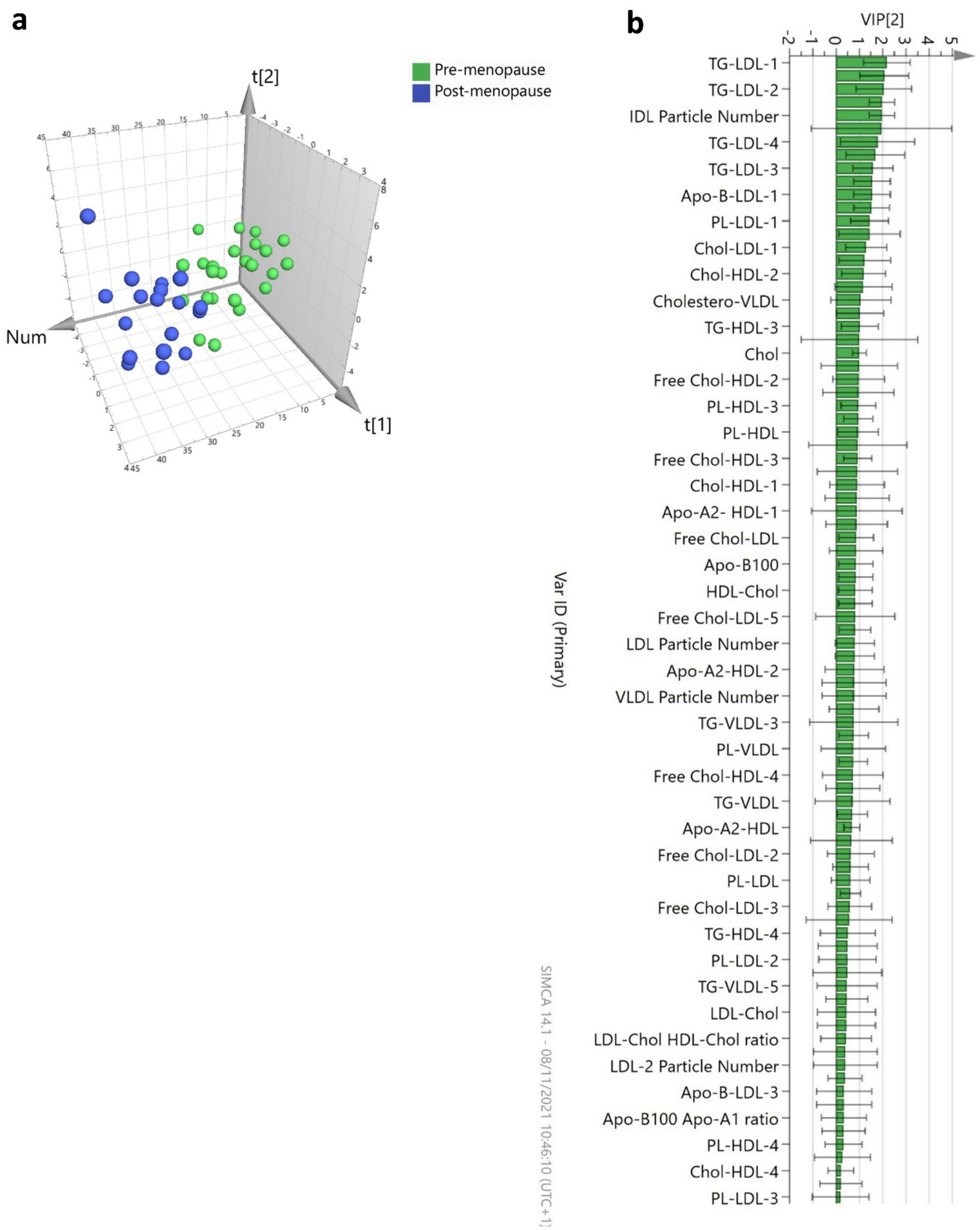

Figure S1. Plasma lipoproteins profiles PLS-DA score plot of HER2- positive BC patients in pre-menopause (n= 25) and post-menopause (n =18) (**a**); Variable importance plot for the projection (VIP) score of the PLS-DA model ranked by increasing values (**b**).

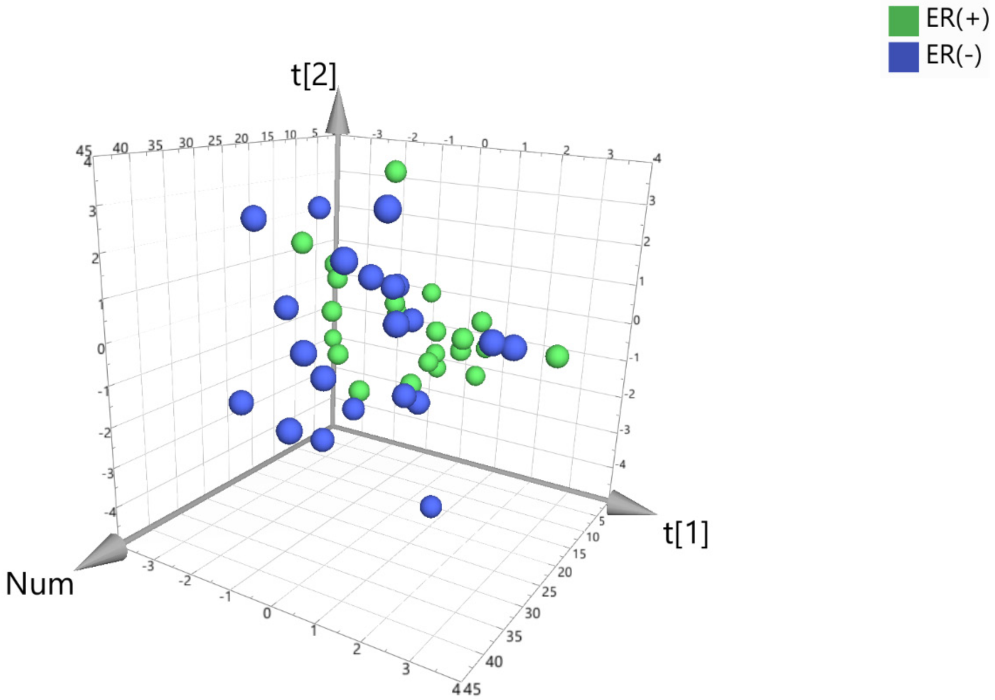

Figure S2. PLS-DA of the plasma baseline lipoproteins'profile for BC patients stratified according to the ER status.
